# Plasma Polymerized Coatings on Hollow Fiber Membranes-Applications and Their Aging Characteristics in Different Media

**DOI:** 10.3390/membranes12070656

**Published:** 2022-06-26

**Authors:** Ashok K. Sharma, Stephen P. Conover, Kamalesh K. Sirkar

**Affiliations:** 1 Applied Membrane Technology, 11558 Encore Circle, Minnetonka, MN 55343, USA; spconover@appliedmembranetech.com; 2 Otto York Department of Chemical and Materials Engineering, New Jersey Institute of Technology, University Heights, Newark, NJ 07102, USA; sirkar@njit.edu

**Keywords:** plasma polymerization, gas separation membranes, nanocoating, aging of plasma polymers, membrane distillation, medical membranes, hollow fiber membranes, ionized air, pore size control, plasma polymerized fluorosiloxane polymer, plasma polymerized siloxane polymer

## Abstract

In the past 30 years, plasma polymerization has emerged as a versatile technique for depositing ultrathin nanocoating on a variety of substrates for applications that range from providing lubricity to the substrate, protection from harsh environments, promoting adhesion, surface modification to applications of coating in ultrafiltration and gas separation membranes. Applications in the field of volatile organic compound (VOC) recovery and membrane distillation have also gained importance in recent years. Most of these applications use silicone and fluorosilicone-based plasma polymers that provide versatility, good separation characteristics, and long-term stability to the membrane. However, plasma polymers are known to age with time. The current study focuses on the aging behavior of silicone and fluorosilicone plasma polymers in different environments that include air, ionized air, heat, aqueous solutions of inorganic chemicals, as well as harsh solvents such as hexane, dichloromethane (DCM), and toluene. Membrane gas permeance and gas selectivity were used to quantitatively measure the aging behavior of the coatings on gas separation membranes, while water and VOC flux were used to measure the effect of aging for membranes designed for membrane distillation and VOC separation. It was found that while all plasma polymers of this study showed changes in membrane gas permeance on exposure to air, they fundamentally retained their membrane separation characteristics in all the studied environments. Significant changes in gas permeability characteristics were observed on exposure of the membranes to organic solvents like dichloromethane, 2-propanol, hexane, and toluene, which are attributed to dimensional changes in the hollow fiber substrate rather than changes in plasma polymer characteristics. Ionized air was also found to have a significant effect on the gas permeability characteristic of the membranes, reducing the gas permeance by as much as 50% in some cases. This is attributed to accelerated oxidation and crosslinking of the polymer in ionized air. XPS studies showed an increase in the oxygen content of the polymer on aging. Differences were found in the aging behavior of polymer coatings made from different monomers with long-chain monomers such as hexamethyltrisiloxane offering more stable coatings. The cross-link density of the polymer also influenced the aging behavior, with the more cross-linked polymer showing a lesser influence on aging in a chemical environment. No significant effect of aging was found on applications of these polymer coatings in the field of membrane distillation, pervaporation, and VOC removal, and a stable performance was observed over a long period of time. It was also noted that the selection of co-monomers played a significant role in membrane distillation, with polymers forming fluoro co-monomers giving better results. The current study also demonstrated the usefulness of plasma polymers in controlling the pore size of microporous membranes that can find useful applications in bio-filtration and VOC recovery.

## 1. Introduction

Plasma polymerization [1,2] has emerged as a powerful technique for depositing ultrathin nanocoating on a variety of substrates. Their use on microporous and nonporous substrates has led to many new applications [3,4,5,6,7,8]. Some of these applications depend on the gas separation characteristics imparted by the semipermeable layer of plasma polymer and some on the surface modification provided by the plasma treatment. For example, the use of plasma polymer-based membranes in lung assist devices, gas separation modules, cell culture, fermentation devices, and analytical chemistry is heavily dependent on the selectivity and flux provided by these membranes [7,8,9,10]. On the other hand, their use in water purification, selective extraction, and other such applications is a result of the surface modification provided by plasma polymerization [11,12,13,14].

A hollow fiber membrane (HFM) coated with an ultrathin nanocoating of a fluorosiloxane (F/Si) plasma polymer was used for direct contact membrane distillation (DCMD). Polypropylene hollow fiber, PP150/330, was found to be the substrate of choice for this application. It offered high water flux and low conductive heat losses due to its larger inner diameter and thicker walls. Plasma polymer coatings brought significant improvements to the performance of these membranes. Water flux in the range of 41–79 kg m^−2^ h^−1^ was reported for 1% saline feed at a feed brine temperature of 60–90 °C. A 5-day long DCMD run under these conditions did not show any evidence of salt leakage into the distillate [15].

Membranes used for VOC removal utilized a thin immobilized layer of a high boiling organic liquid such as silicone 200 fluid in the pores of the Celgard X-10 hollow fibers (ID/OD 240/290 μm, 30% porosity, 0.03 μm nominal pore size) along with an ultrathin coating of siloxane plasma polymer on the membrane surface. Silicone 200 liquid immobilized in the pores of the fiber acted like a liquid membrane and affected the separation. The plasma polymer coating provided stability to the liquid membrane. A separation factor as high as 20 times over nitrogen was reported for these HFMs, depending on the type of VOC and the flow rate of the feed gas used. These HFMs were able to remove up to 99.9% of methanol and toluene at moderate flow rates of 50–100 cm^3^ min^−1^ of the feed gas. The plasma polymer-coated membranes showed extraordinary stability over an extended period of time, ranging from 6 months to two years [16].

A hollow fiber membrane based on PP50/280 fiber coated with an ultrathin nanocoating of fluorosilicone plasma polymer on the outside surface and impregnated with a liquid membrane of tri-n-octylamine was used for removing acetone, ethanol, and butanol from their dilute aqueous solution produced during acetone-butanol-ethanol (ABE) fermentation using the process of pervaporation. Selectivities as high as 275, 220, and 80 for butanol, acetone, and ethanol, respectively, for a feed mixture containing butanol, acetone, and ethanol at the level of 1.5, 0.8, and 0.5 wt.% were achieved [10]. These values are much higher than any value obtained in pervaporation using polymeric or even ceramic membranes and demonstrate the advantage of placing an nanolayer of plasma polymer on the membrane surface to prevent any physical contact between the aqueous feed solution on the outside of the hollow fiber membrane and the immobilized liquid membrane in the pores. The Plasma polymer layer prevented the loss of the liquid membrane as well as contamination of the aqueous feed solution. The liquid membrane of tri-n-octylamine exhibited stability for more than 13 weeks of operation without the need for re-immobilization [17]. The thickness of the plasma polymer coating, as well as the liquid membrane, played a crucial role [18].

The use of plasma polymers in gas separation, dehumidification, gas contactors, diffusers, cell culture, lung assist devices, and ECMO devices have been a subject of interest for the last three decades. These applications typically use X30-240, X30-150, or other similar hollow fiber substrates that have small pore diameters and can be easily converted to a semipermeable membrane by an ultrathin coating of plasma polymer. A hollow fiber membrane based on siloxane polymer has already been commercialized for a lung assist device [7].

The distinction between plasma polymerization and plasma treatment is necessary. While plasma polymerization always leads to the deposition of a layer of polymeric material on the substrate surface, plasma treatment, which is carried out by plasmas of gases such as argon, helium, oxygen, nitrogen, air, ammonia, water vapor, tetrafluoromethane, or blends of these gases, only leads to surface activation, etching, fluorination, amination or surface oxidation. Plasma treatment is often limited to a few atomic layers on the substrate surface [19,20]. However, when any of these gases, except for the inert gases, is used in combination with other monomers, they can copolymerize or increase the rate of deposition or both and enter into the structure of the plasma polymer.

Mechanistic studies suggest that plasma polymerization is a free radical process [21,22,23], although the presence of other energetic species during polymerization cannot be completely ruled out. Radicals are also formed during plasma treatment. It is also known that polymers or surfaces formed by the plasma process age with time, and the aging process leads to the formation of oxygen—functionalities, such as –CHO, C=O, –COOH, –OH –OOH, in the polymer [24]. The aging process can sometimes continue for months and years as the radicals trapped in the bulk of the polymer continue to react and lead to changes in polymer surface functionality, cross-link density, gas permeability, and other properties of the plasma polymer. The extent of change depends on the monomer and plasma conditions used during plasma polymerization or treatment. Conventional polypropylene treated with oxygen plasma or corona discharge, for example, loses its wettability in just a few months. Plasma polymerized propylene, on the contrary, did not lose its wettability after oxygen plasma treatment, even after 12 months of aging [25]. We attributed this difference to molecular overturning on the polymer surface. The changes in mechanical properties of the polymer on aging are expected to be minor due to the extremely thin nature of plasma polymer coatings, (generally less than 0.5 µm or 500 nm), compared to the substrate unless highly reactive oxygen plasma is used for surface modification.

The complexity of the plasma polymerization process makes it difficult to establish the exact mechanism of the aging process, but it is widely believed that free radicals formed during the deposition process get trapped in the bulk of the polymer due to an imbalance between the rate of radical production and consumption and continue to react with atmospheric gases, primarily oxygen. The high cross-link density of plasma polymers results in a tight matrix which slows down the ingress of atmospheric gases to the bulk; as a result, the aging process proceeds slowly and continues for a long period of time.

The atomic nature of the plasma polymerization process adds more complexity and more free radicals to the structure of the plasma polymer, even if the starting monomer has a simple structure [26]. Monomers such as ethylene and propylene do not yield polyethylene and polypropylene like they do in conventional polymerization [27,28]; the plasma polymer formed from these monomers may not be much different from the plasma polymer formed from methane [29] unless very mild conditions are used for the plasma process as in plasma grafting [30,31].

The extent to which the free radicals are trapped in the polymer depends on many factors, but the monomer structure and energy (normally called discharge power) used for polymerization plays the dominant role. Non-functional monomers such as saturated hydrocarbons, silanes, and siloxanes generally result in more trapped radicals. Similarly, high energy used during polymerization leads to more trapped radicals and more aging. This is another reason why non-functional monomers, which typically require higher energy for polymerization, lead to more trapped radicals. High energy also leads to the ablation of the deposited polymer by a process called CAP (competitive ablation polymerization), which competes with the deposition process and results in more radicals [1]. At a very high energy input (W/FM greater than 10^10^ J kg^−1^), the monomer becomes only a source of atoms, and the structural differences between homologs, isomers, and saturated and unsaturated monomers vanish [29]. On the other hand, plasma polymerization conducted at low energy or in a dark region leads to the formation of less trapped radicals [21].

The aging of polymers is not just limited to the air medium. Other media, such as ionized air (as we found in this study), heat, saline solution, alkaline chemicals, organic solvents, and chemicals that are commonly used in membrane application, can accelerate the aging process and limit the use of plasma polymers in many applications. It is, therefore, important to understand how these different media affect the functional properties of the plasma polymer coatings.

Coatings produced from the plasma polymerization of siloxanes and perfluoro monomers are the main focus of this study. Plasma polymerized silanes, siloxanes, and silazanes coatings have been of interest to plasma polymer chemists for many years [32,33,34,35,36]. They offer convenient thin-film alternatives to conventional polysiloxanes, such as silicone rubber, which are versatile polymers and find vast applications in biomedical engineering, electrical engineering, aerospace engineering, automotive applications, O-rings, and many other fields [37] but are difficult to deposit as thin films. Conventional fluoropolymers such as PTFE and PVDF have also been used in many biomedical and engineering applications but are again difficult to deposit as thin coatings. Plasma polymerized siloxane coatings have been successfully used in pacemaker and defibrillator leads for almost three decades. Their use in gas separation is also growing. The plasma polymers based on F/Si monomers have shown great promise in membrane distillation, pervaporation, and fermentation applications [15,16,17,18]. Their use in membrane synthesis has grown several folds in recent years. An understanding of the aging behavior of silicone and fluorosilicone plasma polymers will thus add to our knowledge of the longevity of these polymers in critical applications.

The current paper analyzes the effects of aging of siloxane and fluorosiloxane plasma polymers in different media on the functional properties of hollow fiber membranes without going into the details of the mechanism of the aging process. We have continued to use the term monomers and polymerization to describe the process even though the process does not lead to the formation of polymers in the strict sense, and some researchers prefer to call the product formed residues or coatings. It is for the same reason that the term ‘monomer’ is sometimes referred to as ‘precursor’ by these researchers. However, we have stayed with the conventional terminology that has widely been used in the field of plasma polymerization and used the terms ‘monomers’ and ‘polymers’ in this publication.

## 2. Experimental

### 2.1. Materials and Methods

Silicone monomers in liquid form were procured from Gelest Inc. (Morrisville, PA, USA), and Fluoro monomers were procured from FluoroMed L.P. (Round Rock, TX, USA). Monomers used in this study include: TMDSO (1,1,3,3 tetramethyldisiloxane), TMCTS (1,3,5,7 tetramethylcyclotetrasiloxane), TMSAA (trimethylsilylallylamine), HMTSO (1,1,3,3,5,5 hexamethyltrisiloxane), HFE (hexafluoroethane), PFHP (perfluoroheptane), PFOC (perfluorooctane) and PFHX (perfluorohexane). The inorganic and organic chemicals used for testing resistance and aging of plasma polymer coatings were ACS reagent grade.

Microporous hollow fiber membranes (HFMs) based on polypropylene (PP), namely, X30-240, X30-150, PP50/200, PP50/280, and PP150/330, were used as substrates and procured from Celgard-Membrana or 3M Corporation. Typical properties of the hollow membrane substrates are provided in Table 1.

XPS analysis was used to analyze the composition of polymer coatings. Samples were sent to either EAG Laboratories (Chanhassen, MN, USA) or Anderson Materials Evaluation Inc. (Columbia, MD, USA) for the XPS analysis. FESEM (Field Emission Scanning electron microscopy) was used to examine the surface morphology of the coated hollow fiber membrane. This too was carried out at EAG laboratories.

Scanning Probe Microscopy (SPM) was used to determine the thickness of the coating. This too was carried out at EAG laboratories. AFM (Atomic Force Microscope) images were collected using a NanoScope III Dimension 5000 Digital instrument and processed using the Vision64 software. The gravimetric method was used to determine the thickness of coatings on large size samples.

**Figure 1:**
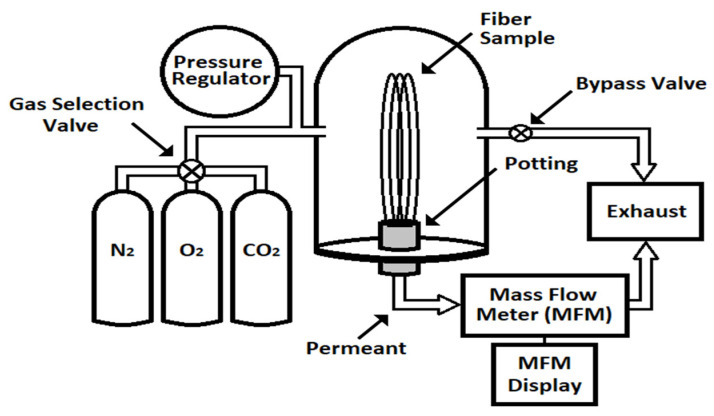
Schematic of the gas permeability apparatus.

Gas permeance, gas selectivity, and water flux through the membranes were used to characterize the functional behavior of the membranes. An in-house built gas permeance apparatus was used for measuring the gas permeance of the membranes [38]. All measurements were made using a differential pressure of 9 psi, except for PP150/330 based membrane, where a lower differential pressure of 3–5 psi was used. A mass flow meter of suitable capacity, made by Unit Instruments Inc. (Yorba Linda, CA, USA) was used to measure the gas flux. Fiber lengths varying from 50–150 cm made into loops of 10–15 cm length placed on a shepherd hook were used for most experiments. All experiments were conducted at a room temperature of 20–25 °C. The schematic of the gas permeability apparatus used is shown in Figure 1.

For aging in air, polymer-coated substrates were left in room air at ambient temperatures of 20–25 °C unless specified otherwise. For aging in liquid media, the samples were immersed in medium of interest for desired length of time and thoroughly washed (with a suitable solvent) and dried after exposure before making the measurements. The effect of aging was studied by testing the gas permeance of the membrane before and after the exposure.

**Figure 2:**
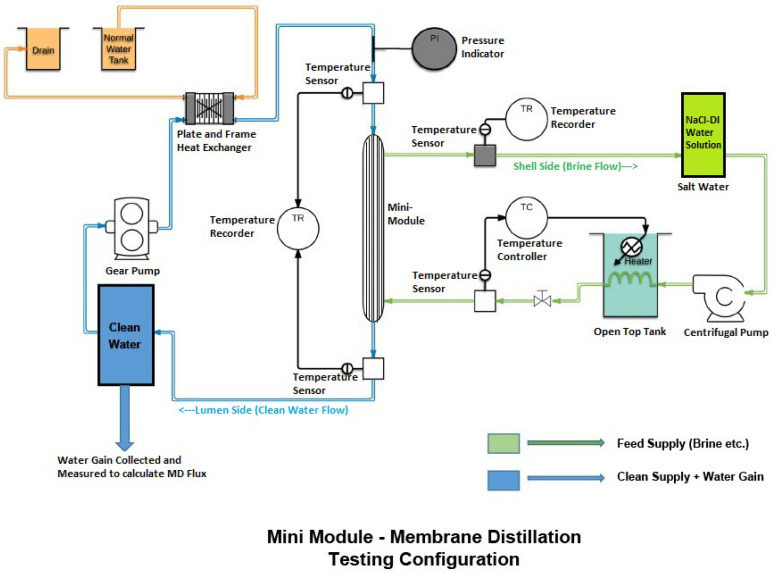
Schematic of the apparatus used for membrane distillation.

An apparatus similar to the one used by Li et al. [15] was used for measuring the water flux in membrane distillation. Modules with a membrane surface area of approximately 52 cm^2^ and a packing density of 12–15% were used for most experiments. The temperature of feed water was varied from 50–81 °C while the cold water temperature was kept at 23 °C. Hot brine was passed on the shell side of the membrane, and a stream of cold water through the lumen of the fiber which collected the permeated water. The details are provided in the reference quoted above. The schematics of the apparatus used are shown in Figure 2.

A Simco Ionizing air blower (Model Aerostat XC, Hatfield, PA, USA) was used for aging the membranes in ionized air.

Contact angle measurements were made on plasma polymer coated micro-glass slides using a Rame–Hart goniometer (Model 100–00-115). A Gilmont glass syringe was used for depositing drops of distilled-deionized water on the substrate surface.

### 2.2. Plasma Polymerization Process

**Figure 3:**
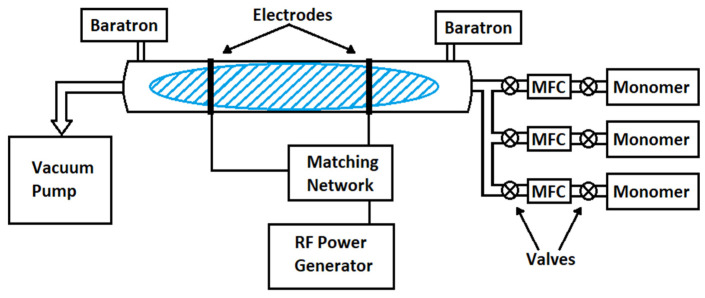
Schematic of Plasma Polymerization Reactor.

A tubular reactor with capacitive coupling operating at 13.56 MHz RF energy was used for plasma polymerization. The schematic of the batch reactor used are shown in Figure 3. Plasma polymerization was carried out either in a batch reactor or in a continuous reactor at low pressures of 10–200 mtorr using 10–200 Watt plasma energy. An outline sectional view of the continuous plasma polymerization reactor used in this study is given in reference [39]. The electrodes were connected to an RF power supply (Model RF-5S made by RF Power Products Inc. (Vorhees, NJ, USA) through a matching network of capacitors and condensers. The flow of monomer(s) in the reactor was controlled using mass flow controllers (model UFC 1200 and UFC 1100 made by Unit Instrument Inc. (Yorba Linda, CA, USA). The pressure in the reactor was measured using Baratron pressure gauges (Model # 220DA-00001B2B) made by MKS Instruments Inc. (Andover, MA, USA). Heat was used if the monomer was unable to generate enough vapors at room temperature. Inorganic gases such as oxygen, nitrogen, and argon were sometimes used for the activation of the substrate or as a co-reactant in the plasma process.

The reactor was pumped down to less than 10 mtorr before the monomer(s) were introduced in the reactor, and the pressure was allowed to stabilize. Substrate samples were either hung in the reactor or passed from reel to reel in a continuous fashion through the plasma zone. The plasma was initiated by applying RF power to the capacitively coupled electrodes placed outside the reactor chamber. The thickness of the coated membrane was controlled by varying the residence time (RT) of the substrate in the reactor or by changing the plasma energy or monomer flow rates or all of them. More details on the reactor and plasma polymerization process can be found elsewhere [39,40].

Liquid membranes for selective VOC separation, referenced in this paper, were prepared by impregnating the Si or F/Si polymer-coated HFM with a high boiling organic liquid such as TOA (trioctylamine) or DC 200 Silicone fluid. The height of the liquid in the pore was adjusted either by flushing the impregnated liquid membrane with a solvent or by using a dilute solution of the impregnating liquid in ethanol or hexane. The gas permeance of N_2_ was used to determine the adequacy of the liquid membrane thickness for the intended application. For details, see references [10,17]. The hollow fiber membranes for VOC removal were made using X30-240 hollow fiber as the substrate, while for alcohol-water separation, PP50-280 substrate was used. The coating consisted of F/Si or Si- plasma polymer. VOC experiments were carried out at NJIT and are presented here only for reference purposes [16,18]. Membrane distillation experiments were carried out using PP150/330 HFM coated with a F/Si polymer coating.

## 3. Results and Discussion

### 3.1. Pore Size Control Using Plasma Polymerization

Since plasma polymerization is a gas phase process and polymer build-up on the substrate occurs in a layered manner, the technique has been successfully used for controlling the pore size of microporous HFMs. This is demonstrated in Table 2 and Table 3 for PP 50/280 and PP 50/200 HFMs respectively and in Table 3 for PP150/330 HFM. The N_2_ permeance of the HFMs is gradually reduced by increasing the amount of polymer forming monomer (PFM), TMDSO, in this case, in the feed.

Liquid flow through a porous medium is often described by Darcy’s equation. The relation for gas flow through larger pores follows the Poiseuille equation for a compressible fluid; for smaller pores under suitable conditions, Knudsen flow is applicable. Under conditions of Poiseuille flow of a compressible fluid, the relationship between the pore structure and permeability k is given by the following proportionality relation:

k ∝ c ϕ r^2^ (1)

where ϕ is the porosity, r is the pore radius, and c is the geometric factor that accounts for the shape, connectivity, aspect ratio of pores, and tortuosity of the pores. For liquid systems, the proportionality may be replaced by an equal sign in Equation (1); for gaseous systems, permeability k depends on pressure conditions on the two sides (see, Sirkar, 2014) [41].

If it is assumed that the Poiseuille equation is applicable to the current membrane system, the decrease in N_2_ permeance is proportional to the square of the change in the effective diameter of the pores. Table 2, Table 3 and Table 4 also provide an estimate of the effective pore diameter of the coated HFM, assuming that Equation (1) is applicable and the pore diameter of the untreated HFM is 0.2 μm for PP50/200 and PP50/280 HFM and 0.6 μm for PP150/330 HFM. The monomer flow rate and residence time of the substrate in the plasma reactor both played a role. Higher monomer flow rate and longer residence time both led to a decrease in gas permeance and effective pore diameter.

A similar effect on gas N_2_ permeance was observed for PP50/200 hollow fiber membrane, which has a smaller inside diameter than the PP50/280 fiber. The effect of PFM concentration was even more pronounced for this membrane, as seen in Table 3. The reason for a sharp decrease in gas permeance of the treated PP50/200 membrane compared to the PP50/280 membrane, on increasing the PFM concentration, in spite of their similar pore size diameter, is not clearly known. The difference in porosity or the pore size distribution or both could be the reason. The membrane used in sample nos. 2417, 2418, and 2420 were semipermeable in nature and presented an O_2_ to N_2_ selectivity of 1.14, 1.30, and 1.36, respectively, indicating that the pores in these membranes were almost closed.

The effect of changing the PFM concentration was much less pronounced for PP150/330 HFM, as seen in Table 4. It is partly because of the larger pore size of the PP150/330 hollow fiber. The membrane remained widely open in spite of a decrease in pore diameter due to plasma polymer coating.

The findings are along expected lines. Slow closing of pores is expected since plasma polymerization is a gas-phase reaction. Complete closing of pores for PP50/280 and PP150/330 hollow fiber membrane requires considerable effort, and hence these membranes, especially the PP150/330 fiber, are not suited for gas separation application. Moreover, these membranes do not show any significant change in gas permeance as a result of aging. Most aging experiments in our study were, therefore, performed on an X30/240 hollow fiber membrane, which has an extremely small pore size, and even a thin coating of plasma polymer closes the pores resulting in a semipermeable membrane.

### 3.2. Thickness of Plasma Polymer Coatings

The uneven surface of the hollow fiber membrane and ultra-low thickness of plasma polymer coatings made it difficult to accurately measure the thickness of plasma polymer coating by optical methods, including SPM and SEM. The measurements were therefore carried out on solid silicone tubing substrates, but even then, a significant unevenness across the coating was observed, which reduced the accuracy of the thickness measurement. The average thickness measured by SPM on two separate samples were 472 and 470 nm. However, there was a high amount of uncertainty in the average thickness as both samples had very high and low point extremes resulting in thickness variation between 8.70 nm and 1000.13 nm. In spite of large variations, the average values were surprisingly close to the thickness that we measured by the gravimetric method on a large size sample of silicone tubing coated under similar conditions. The gravimetric method gave us an average value of 420 nm for thickness. Since the coatings on fiber samples were produced at a shorter residence time, we expect the thickness of the coatings on fiber samples to be in the range of 50–400 nm.

### 3.3. Results of Field Emission Scanning Electron Microscopy (FESEM)

**Figure 4:**
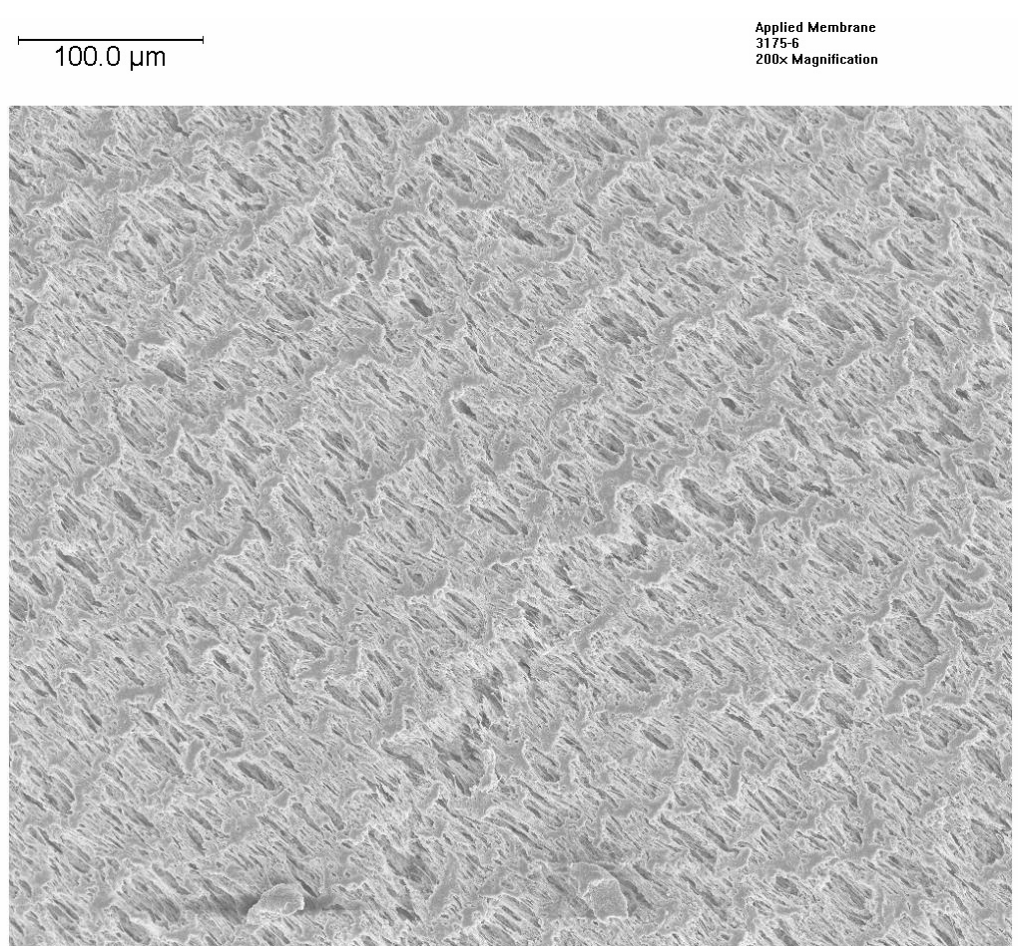
SEM of Plasma Polymer Coated PP150/330 HFM.

**Figure 5:**
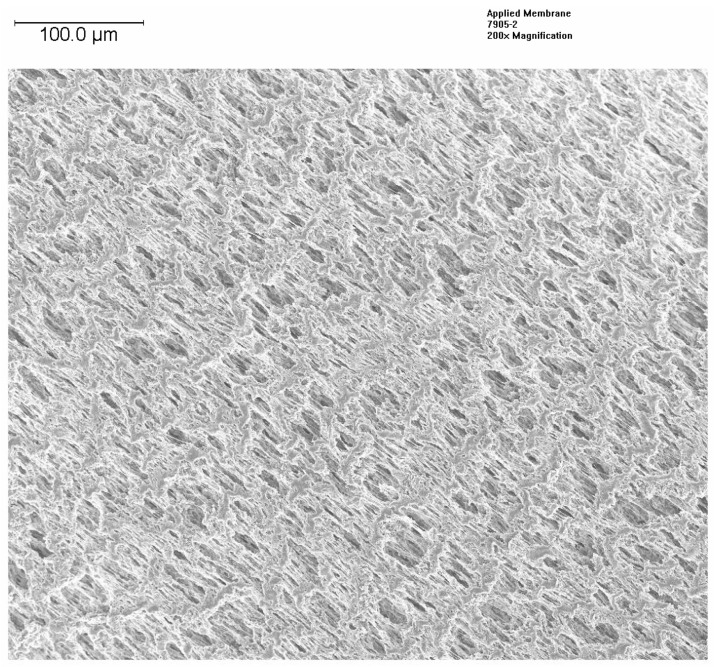
SEM of uncoated PP150/330 (7905 reflects the batch of uncoated membrane).

Changes in the morphology of the HFM were examined by FESEM at EAG Laboratories. The samples were coated with an ultrathin layer of platinum to reduce charging. Figure 4 presents the SEM images of a coated PP150/330 fiber with a plasma polymer having a composition similar to sample 5803, while Figure 5 presents the image of the same fiber before coating. As one can see, the porous structure is retained after the coating, although the size of the pores appears to be reduced. This is in agreement with the results presented in Table 4 for PP150/330 membrane. Thongsukmak and Sirkar observed a similar effect on the PP50/280 membrane, that was heavily coated, in their study [10].

### 3.4. Contact Angle with Water

Both siloxane and fluorosiloxane plasma polymer coatings demonstrated considerable hydrophobicity, as indicated by the contact angle with water. The results in Table 5 were obtained by coating the plasma polymers on pre-cleaned microglass slides. An average of three measurements is presented for each sample. No effect of aging in the air was noted on the water contact angle of these coatings, indicating that the coating’s performance in membrane distillation or other such applications will not change as a result of aging in air. No change in contact angle with water was observed even after 3 years. The water repellency in the case of fluorosilicone polymers was retained even after 48 h immersion in distilled water. Short-chain perfluoro monomers such as HFE produced polymer coatings of poor structural integrity when plasma polymerized but produced hydrophobic coatings of good structural integrity when copolymerized with siloxane monomers. The results also confirm that the changes brought by aging do not significantly affect the surface energy of the plasma polymer coatings.

Similar results were reported by Song et al., who studied the contact angle on PP50/280 and PP150/330 hollow fibers before and after coating with a fluorosiloxane plasma polymer [42]. A Cahn balance (DCA 315, Thermo Fisher Scientific, Waltham, MA 02454, USA) was used to characterize the dynamic contact angle of water on hollow fiber membrane surfaces in this study. A membrane prepared in Experiment 5803 was used for these experiments. Advancing contact angle of as high as 143° with water was observed for the plasma polymer coated PP150/330 fiber compared to 100 °C for uncoated fiber membrane.

### 3.5. Tensile Properties

Tensile properties were studied using Instron Model 112 Tensile tester. The tests were carried out at room temperature (20–25 °C) according to ASTM D3822-91. The 500 g load cell and a special pneumatic grip for holding the fiber samples were used. The single fiber was tested at a crosshead speed of 25 cm min^−1^. A gauge length of 5 cm was used to accommodate for the high elongation of the fiber samples.

Table 6 lists the typical tensile properties of PP 50/200 hollow fiber membranes before and after coating with a plasma polymer made from TMDSO. A non-polymer forming fluoro monomer (HFE) was used as a co-reactant. As one can notice, there is no significant effect of plasma polymer coating on the tensile properties of the fiber. The breaking load and elongation stayed in the experimental error range. Using a higher amount of TMDSO in the feed did not bring any significant change in tensile properties, although the plasma energy parameter W/FM changed. This is expected because the polymerization was carried out at a low plasma energy of 80 W and the thickness of the coating was less than 0.4 µm (400 nm) which is less than 1% of the wall thickness of the hollow fiber substrate and less than 0.3% of the overall cross-section. The higher standard deviation within the samples can be attributed to the shorter fiber length used in these studies. The shorter lengths of the specimen can decrease the accuracy of the tensile properties determined, according to the method. W/FM is a composite plasma energy parameter, where W is the RF power used for polymerization, F is the monomer flow rate, and M is the molecular weight of the PFM.

Results for X30-240 hollow fiber membranes prepared by coating with a plasma polymer made from TMDSO are listed in Table 7. In this case, the tensile strength of the fiber increased as a result of plasma polymer coating, and % elongation decreased. Once again, no significant effect of the varying plasma energy (W/FM) was observed. The sample length in this study was kept at 15 cm, as suggested in the method. The standard deviation within samples was low. Membranes based on X30-240 were designed for gas separation applications, and the plasma polymerization conditions used were different. The resulting membranes were semipermeable and had no physical pores left, as suggested by their gas selectivity. The complete filling of the membrane pores resulted in more rigid membranes which had higher tensile strength and lower elongation.

### 3.6. Development of Membranes for VOC Removal, Pervaporation and Direct Contact Membrane Distillation (DCMD)

Plasma polymerization has emerged as a powerful technique for the development of highly selective hollow fiber membranes for VOC removal, pervaporation and direct contact membrane distillation (DCMD), an emerging technique for water purification [43]. Some of the earlier work by this group was covered in the introduction. Further work in our laboratory has shown that using a ‘polymer forming fluoro co-monomer’ (PFFCM) such as PFHX, PFHP, and PFOC in DCMD for the synthesis of the F/Si copolymer, instead of a non-polymer forming fluoro monomer (NPFFCM) such as CF_4_ and HFE, along with the polymer forming silicone monomer yields the best results in DCMD. These results are summarized in Table 8. Parallel flow modules based on PP150/330, with a packing density of 10–15% and HFM surface area of 51.9 cm^2^, were used for these experiments. Notice the higher flux obtained with the polymer forming fluoro co-monomer. Some of the membranes maintained their performance over a test period of 3 months. The higher concentration of fluoro-moieties resulting from polymer forming fluoro monomer in the coating compositions led to an improvement in the thickness and hydrophobicity of the hollow fiber membrane, which in turn led to higher water flux and provided better stability to the membrane. The water flux results are comparable to those reported by Sirkar et al. in spite of significant differences in module geometry [15].

Table 9 presents the effect of brine temperature on the water flux through these membranes. Notice the exponential increase in the water flux as the feed temperature was increased to 81 °C. The results confirm how significant a role the brine temperature can play in the DCMD process.

No significant effect of the aging of polymer coating was seen for these membrane applications. The water flux results for membranes aged 2 and 3 months were comparable to the freshly made membrane. See the results for sample no. 6199 in Table 8. Similar results were reported by Sirkar et al. in their studies of plasma polymer-coated membranes made by this group for VOC recovery and are presented in detail in reference [18]. The separation phenomenon in VOC removal, membrane distillation, and liquid membrane-based pervaporation are dependent on surface energy and pore size of the membrane, and none of these properties are expected to be significantly affected by the aging of plasma polymer.

### 3.7. Semipermeable Membranes from Plasma Polymerization and Their Aging Characteristics in Air

Plasma polymerization has proven to be a very effective technique for producing semipermeable membranes useful for gas separation. The plasma process does not use any solvent and is carried out in a vacuum under clean room conditions. The membranes produced often show high gas flux and selectivity due to their extremely low thickness and are expected to be resistant to body fluids as well as strong chemicals used in chemical processing. The thermal and mechanical stability of the membranes are limited only by substrate characteristics as plasma polymers have very high thermal stability showing no significant degradation even at 300 °C in air and 600 °C in an inert atmosphere for plasma polymerized methane [29]. Since coating thickness is seldom greater than one μm, the substrate’s mechanical properties also remain unaffected. As a result of these unique features, plasma polymer coated gas separation membranes are finding application in dehumidification, CO_2_ removal, artificial lungs, and extracorporeal medical oxygenator (ECMO) devices [44,45]. The substrate used for synthesizing these membranes typically has a small pore size, less than 0.1 μm, and preferably less than 0.05 μm. As a result, microporous substrates such as X30-240 and X30-150 are considered the substrates of choice for gas separation applications. Novel membranes that use monomers of higher molecular weight, such as HMTSO and OMTSO (1,1,3,3,5,5,7,7 Octamethyltetrasiloxane), offer a special advantage. The gas flux and membrane utilization factor (MUF), which is a composite parameter of gas selectivity and permeance, for these membranes are higher than membranes synthesized from simple monomers such as TMDSO and TMCTS [40]

The aging of plasma polymers, which did not affect DCMD applications, can, however, be bothersome for gas separation applications. Table 10 shows the effect of aging of membranes made from TMDSO, TMCTS, TMSAA, and HMTSO in air at different time intervals. As can be seen, the biggest change in gas permeance occurred in the first 7 days of membrane synthesis. The permeance of gases N_2_ and O_2_ (not reported here) also decreased.

**Figure 6:**
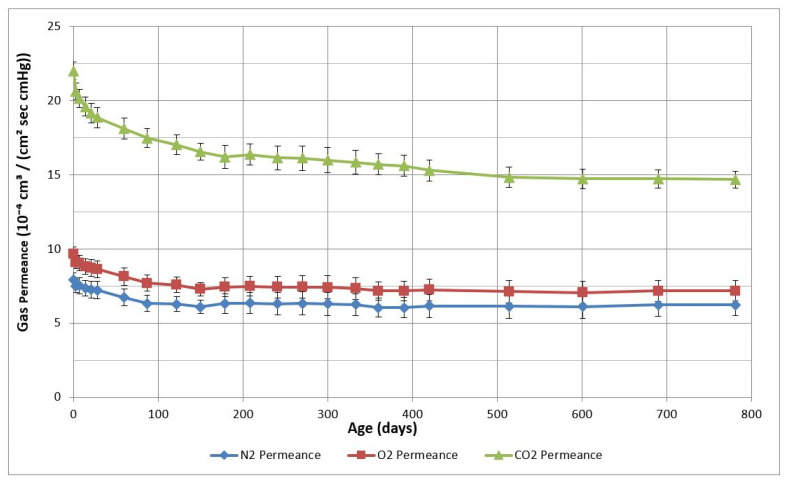
Effect of aging of TMDSO membranes on shepherd hooks in room air on the gas permeance for N_2_, O_2_, and CO_2_ gases.

Figure 6 shows the changes in the N_2_, O_2,_ and CO_2_ permeance of semipermeable membranes prepared from TMDSO monomer over a period of 800 days. Membranes in this study were tested and aged on shepherd hooks and fully exposed to room air. The average values of permeance are presented. The sharp change in permeance in the initial stages of aging is noticeable for all gases. Not much change is noticeable after about 450 days. An upward trend in gas permeance for all gases at about 200 days was because of a change in the gas flow meter.

The polymers made from structural monomers, such as TMSAA, in general showed less change in gas permeance on aging. This is partly because these monomers can be polymerized at low plasma energy, resulting in a lower concentration of the trapped free radicals. The molecular weight of the PFM also played an important role, with higher molecular weight monomers, such as HMTSO, producing less change in gas permeance. This is believed to be due to the higher polymer forming capabilities of larger molecular weight monomers, which generate less fragmentation and hence fewer trapped radicals [40].

That the aging process involved the interaction of the trapped radicals with atmospheric oxygen was demonstrated by testing the interior layers of the coated membranes on the spool. The results are shown in Table 11 for a TMDSO Plasma polymer-coated X30-150 HFM. The membrane in the interior of the spool showed much less change in gas permeance. The CO_2_ permeance for these membranes decreased only by 4.1% over a period of 10 years. There was virtually no change in the CO_2_/O_2_ selectivity of the membrane. It appears that the free radicals trapped in the polymer coating in the bulk of the membrane spool lost their spin through intermolecular reactions or cross-linking rather than oxidation that is dominant at the surface of the spool, which can produce a more drastic effect. The intermolecular reactions can make the membrane tight for all three gases due to changes in the gas diffusion coefficients. On the other hand, oxidation of polymer can lead to varying effects due to differences in the solubility coefficient of the gases. Even the effect of cross-linking can vary as the CO_2_ molecule has the smallest kinetic diameter of the three gases. The overall effect of aging can thus be complex and difficult to interpret. XPS studies on Plasma polymerized methane showed less reacted oxygen in the bulk of the polymer than on the surface [29]. Aging under an N_2_ atmosphere also slowed down the drift in gas permeance, confirming the role of O_2_ in the aging process.

Similar results were obtained on TMDSO polymer-coated X30-240 HFM. Table 12 compares the gas permeability data for polysiloxane coated X30-240 HFM aged on a shepherd hook and on a spool. As can be seen, aging on shepherd hooks for 515 days (around 17 months) resulted in more changes in gas permeance as well as the gas selectivity for all gases. The same membrane on a spool, when tested after 4650 days (around 12 years) after removing approximately 500-m of membrane from the top layer, showed much less change in gas permeance and gas selectivity. Although the CO_2_ flux was reduced by approximately 23%, the selectivity of HFM showed no change even after 12 years. The shepherd hooks made from the 12-year-old HFM showed much less decay in gas permeance after further aging for 6 months, indicating that most spins were neutralized, although small changes still continued. There was virtually no change in N_2_ and O_2_ gas permeance, and the CO_2_ permeance got reduced by only 5.6%. This is much smaller compared to the 22% reduction observed just after 4 months for the fresh membrane (see Table 10). Progressive hardening of the potting compound and changes in lab humidity or temperature may also contribute to these changes.

### 3.8. XPS Data

**Figure 7:**
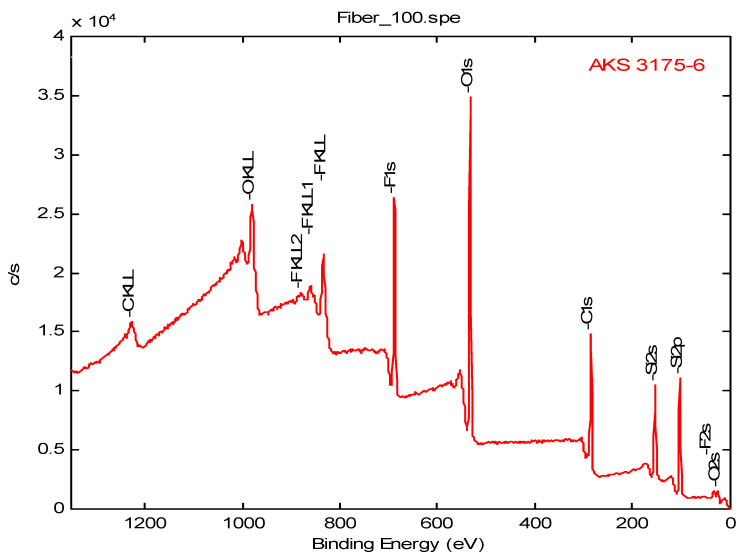
XPS spectrum of the F/Si plasma polymer coated PP150/330 membrane.

**Figure 8:**
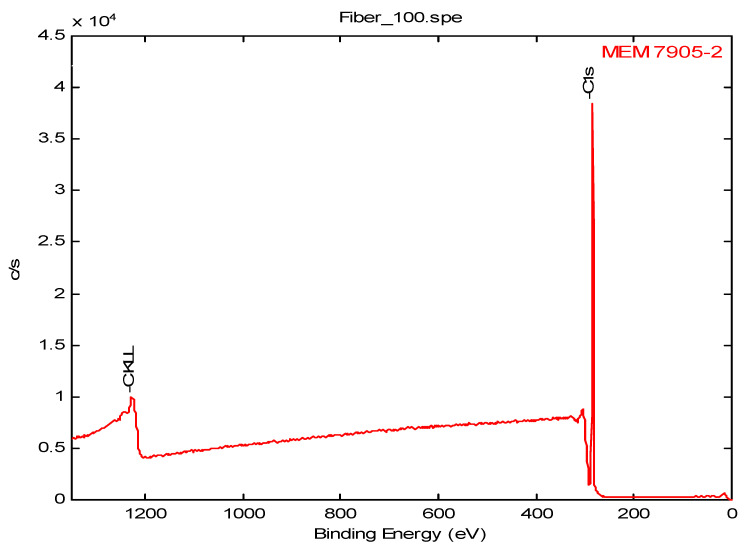
XPS spectrum of the Uncoated PP150/330 membrane.

The XPS analysis was performed over an elliptical area irradiated by the low energy (1487 eV) monochromatic aluminum Ka X-ray with a major axis of 1.0 mm and a minor axis of 0.5 mm. The atomic concentration of elements found near the surface region of the TMDSO plasma polymer coated X30-240 aged for 33 and 52 days are presented in Table 13. The elemental composition indicated considerable de-carbonization of the siloxane monomer used during polymerization. The C/Si ratio dropped to 1.39 from 2.0, while the O/Si ratio increased to 1.23 from 0.5. The increased amount of oxygen in the coating compared to the monomer indicates surface oxidation of the polymer resulting from the interaction of residual radicals on the surface with the atmospheric air. The O/Si ratio further increased on aging of the membrane. These results are consistent with the results reported in our earlier work [29,46]. Table 13 also shows the elemental composition of a F/Si plasma polymer (#3175) coated on a PP150/330 substrate along with that of uncoated fiber (#7905). Figure 7 and Figure 8 show the XPS spectrum of the F/Si polymer coated and uncoated PP150/330 HFM, respectively. The data for the uncoated HFM (#7905) is typical of a hydrocarbon polymer, although the low-resolution spectrum was not able to resolve the valence bonds to the degree necessary for a more positive identification of the polymer type. The XPS spectrum of the F/Si plasma polymer coated fiber (#3175) showed distinct peaks for Si, F, and O besides that of carbon.

### 3.9. Heat Aging

As shown in Table 14, semipermeable membranes prepared from TMDSO plasma polymer, when heat-treated at 90–100 °C for a period of 90 min, showed a reduction in gas permeance of approximately 15–20% for CO_2_ depending on the thickness of the membrane used. The permeance of O_2_ and N_2_ (not shown here) dropped by 20–25%. Membrane based on HMTSO showed an even bigger drop (29%) in CO_2_ gas permeance and approximately 35% drop in O_2_ and N_2_ permeance. This reduction in gas permeance is partly due to the longitudinal shrinkage in the polypropylene HFM as a result of the heat treatment and partly due to increased cross-linking of the plasma polymers. No visual change in the diameter of HFM was noticed. When the correction for the change in ‘length alone’ of the membrane was applied, the change in CO_2_ gas permeance for HMTSO plasma treated membrane dropped to 16.6%, which was lower than the 29% observed without the length correction. The CO_2_/O_2_ selectivity increased in almost all cases, while the O_2_/N_2_ selectivity (results not shown here) remained mostly unaffected or increased by approximately 5% for the HMTSO membrane. The HFM samples used in this study were approximately 2 years old. Heat aging at a lower temperature of 55–60 °C (results not shown) showed very little change in gas permeance or selectivity even after 96 h.

### 3.10. Aging in Contact with Chemicals

Most inorganic chemicals or dilute solutions of organic chemicals tested in this study showed no significant negative effect on the gas selectivity of the membranes prepared by plasma polymerization, although gas permeance was reduced in almost all cases.

#### 3.10.1. Aging in Buffered Saline

Table 15 shows the effect of aging on the gas permeability characteristics of the TMCTS plasma polymer coated X30-240 hollow fiber membranes exposed to 0.9% buffered saline solution at 37 °C for an extended period of time ranging from 7–28 days in order to assimilate physiological conditions since some of the membranes of this study were designed for biomedical applications. The samples were washed with distilled water and dried overnight at 40–50 °C in an oven after each exposure. Three samples were tested in each case to check the reproducibility of the results. The gas selectivity marginally improved, and gas permeance marginally decreased for all samples. The integrity of membranes was, however, retained in all cases. The overnight drying of test samples in oven, partial retainment of solvent in the dried samples, or changes in test conditions can be the reason(s) for the change in gas permeability characteristics. Table 16 shows a similar effect of aging in 0.9% buffered saline for TMDSO plasma polymer coated X30-150 hollow fiber membrane. Here a slight, nonsignificant decrease in CO_2_/O_2_ selectivity was observed.

#### 3.10.2. Aging in Contact with Alkaline solutions and Organics

Table 17 shows the effect on the gas separation characteristics of a TMDSO plasma polymer coated X30-240 membrane after exposure for 20 h at room temperature to a host of other chemicals that include 0.45 M and 0.90 M NH_4_OH solutions, 20% aqueous solution of diethanolamine (DEA), 50% solution of TOA in Ethanol (EtOH), 50% solution of DC 200 silicone fluid in ethanol, hexane, 2-Propanol and dichloromethane (DCM). The samples were washed and dried with pure solvent and dried in an oven at 40–50 °C overnight, after each exposure, before they were tested again. While NH_4_OH solutions had very little effect on the gas selectivity, and gas permeance decreased only by 7–8%, exposure to organic chemicals led to a significant effect on the gas separation characteristics of the membrane. While gas permeance decreased in all cases, the selectivity increased after exposure to 2-Propanol and DCM. The results with dilute solutions of organics and hexane were intermediate. While CO_2_/O_2_ selectivity decreased by 8.7% after exposure to hexane, it changed only by 2–5% for solutions of DEA, DC 200, and TOA. The gas permeance and selectivity marginally recovered after further drying of the membrane in the case of TOA, indicating that entrapment of chemicals in the pores may also be partly responsible for the change in permeability characteristics. The reason for greater changes in exposure to 2-Propanol and DCM is not clear, but the stress and changes in pore structure caused by swelling and de-swelling of polymeric membrane may be the reason. Nevertheless, the membranes did not lose their integrity and remained semipermeable after exposure to all tested chemicals.

Table 18 depicts the effect of change in the composite plasma energy parameter W/FM on the change in the gas permeability characteristics of a relatively thick polysiloxane membrane, prepared from TMDSO monomer by increasing the reaction time and PFM flow rate, on exposure to hexane and toluene solvents (see Section 3.5 on tensile strength for an explanation of the W/FM parameter). As the results indicate, the membrane prepared at higher W/FM produced a lower drop in gas permeance and selectivity due to higher cross-link density which led to less irreversible swelling of the membrane in contact with hexane and toluene.

**Figure 9:**
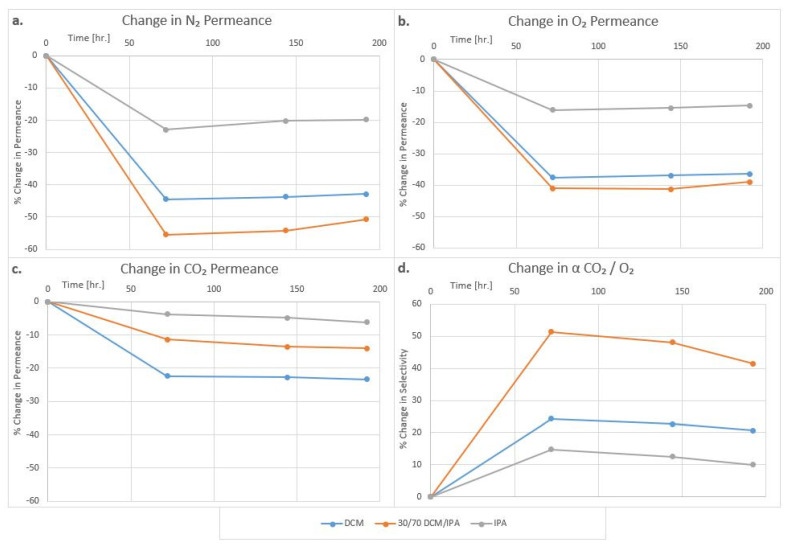
Effect of storing TMCTS plasma polymer coated HFM in DCM, DCM/IPA, and IPA on N_2_ (**a**), O_2,_ (**b**) and CO_2_ (**c**) gas permeance and CO_2_/O_2_ selectivity (**d**).

The result of exposure to dichloromethane (DCM) and 2-propanol (IPA) are also illustrated in Figure 9. The membranes for this study were made from TMCTS plasma polymer coated on X30-240 HFM. Even a 15-min exposure of HFM to DCM led to a 22% drop in CO_2_ gas permeance and a 42% drop in O_2_ gas permeance. The recovery was less than 5% even after 200 h. In contrast, 2-propanol (IPA) produced less than 5% change in CO_2_ gas permeance and approximately 15% change in O_2_ and N_2_ permeance. The results with the blend of two solvents were intermediate. The membrane integrity was not affected in either solvent, and all membranes remained permselective. The CO_2_/O_2_ selectivity became even higher after exposure to these solvents. The results show that the plasma polymer coatings have excellent resistance to inorganic and organic chemicals. This is not surprising as plasma polymers are known to have excellent chemical resistance due to their chemical structures and high cross-link density, and the current results confirm earlier findings [1]. Thus, the change in gas permeability characteristics must be a result of the change in the dimensions of the substrate membrane caused by swelling and de-swelling of the PP-based hollow fiber leading to tightening of the membrane structure. The retainment of minute traces of chemicals in the membrane pores and changes occurring as a result of drying in the oven cannot be completely ruled out.

Microscopic examination of the knitted mats made from TMCTS plasma polymer coated X30-240 hollow fiber membrane before and after 15 min exposure to DCM led to approximately 20% reduction in mat density (HF/cm), which was irreversible. This confirms that the drop in the gas flux is at least partially a result of the change in surface area of the membrane and the change in gas selectivity, a result of the change in diffusion coefficients of gases through the modified polymer structure.

### 3.11. Aging in Ionized Air

**Figure 10:**
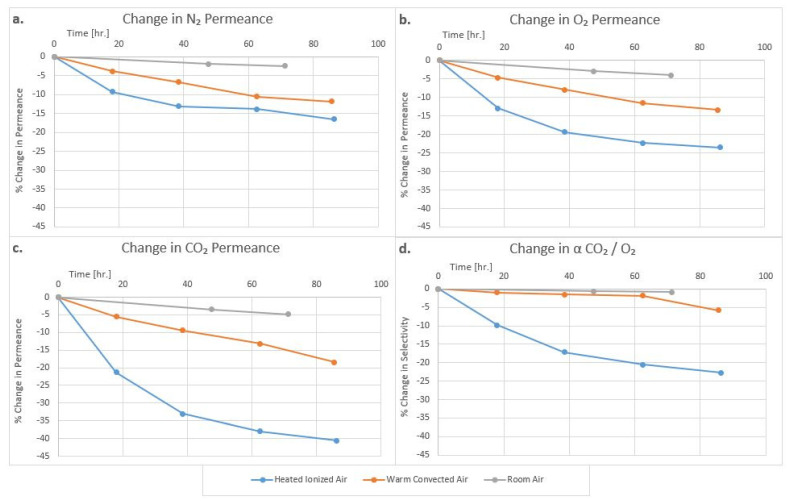
Effect of heated Ionized, warm convective air and room air on N_2_ (**a**), O_2,_ (**b**) and CO_2_ (**c**) gas permeance and CO_2_/O_2_ selectivity (**d**) of TMCTS plasma polymer-based membrane.

Perhaps the most dramatic effect of aging of plasma polymerization-made membranes on gas permeability characteristics was seen when the samples were aged in an ionized atmosphere, e.g., in air blown from an ionizing air blower typically used for removing static charges from substrates. A Simco Ionizing Air blower (Model: Aerostat XC) was used in our study. In this case, a sharp drop in gas permeance was observed within a short span of time. The highest drop came in the first 48 h. The CO_2_ gas permeance dropped by as much as 35%, and the N_2_ gas permeance dropped by approximately 14%. The O_2_ gas permeance dropped by approximately 22%. The sharp drop in CO_2_ permeance compared to other gases also reduced the CO_2_/O_2_ selectivity by as much as 18% in 48 h. The change in gas permeance brought by ionized air was irreversible. The results are shown in Figure 10 for membranes based on TMCTS plasma polymer. No significant difference was observed in cold vs. hot ionized air. These findings are significant as ionized air is commonly used in membrane module preparations to reduce electrostatic charges and facilitate bundling. No change in membrane diameter was noted.

The reason for this dramatic change in gas permeability characteristics on exposure to ionized air is not clearly understood. It is believed that the ionized air leads to rapid oxidation and cross-linking of the membrane both at the surface as well as in the interior of the membrane resulting in the formation of oxygen functionalities and a tight membrane structure which leads to a drop in the gas selectivity and permeance due to change in diffusivity and solubility parameters of the membrane.

Figure 10 also shows the effect of aging of the plasma polymer membranes in stagnant room air and warm air (30–35 °C) convected from a hot air blower for the same length of time. In both cases, the drop in the gas permeance was significantly lower than that in the ionized air. The change in CO_2_ gas permeance in the stagnant room air at room temperature, which represents the natural aging of the membrane, was approximately 4% in 48 h. The change in N_2_ permeance was approximately 3% in 48 h. The CO_2_/O_2_ selectivity was reduced by approximately 1% over this period of time. The effect of aging in warm convected air was higher than in stagnant air at approximately 10% for CO_2_ gas, but it was still much less than the ionized air. The warm flowing air also accelerated the oxidation of the membrane but at a slower rate than the ionized air. The change in CO_2_/O_2_ selectivity was approximately 2% over a period of 48 h. The membranes used in this study were already room temperature aged and a few months old.

## 4. Concluding Remarks

Plasma polymerization finds application in both industrial and biomedical engineering. Gas separation membranes made using plasma coatings have been used in novel extracorporeal membrane oxygenation (ECMO) and lung dialyzer devices. The F/Si polymer coating produced by plasma polymerization has shown promising results in membrane distillation, VOC recovery, and separation of fermentation products. This paper reports yet another application of plasma polymerization; its use for membrane pore control, which can have significant potential in biochemical fields. We were able to gradually reduce the pores of microporous PP50/200 and PP50/280 hollow fiber membranes by careful control of plasma conditions.

Plasma polymers are, however, known to age through the chemical reaction of trapped radicals with atmospheric oxygen and intermolecular cross-linking. This study has analyzed the effect of aging on the gas permeability characteristics of the hollow fiber membranes prepared from X30-240 and X30-150 HFMs by applying a thin layer of plasma polymer made by plasma polymerization of a siloxane monomer. Different aging media, which included chemicals besides air, were used. The results indicated that the aging process is gradual and slows down as time proceeds. The aging can be accelerated by forcing air into the membrane interior, which besides oxidation of the membrane, also leads to changes in the cross-link density in the bulk of the polymer. Ionized air leads to a rapid change in the membrane gas permeance by providing reactive species, which accelerates the oxidation process. Care should, therefore, be taken and allowances made for the aging effect while designing devices which are heavily dependent on the gas permeability and selectivity of the membranes. The mechanical integrity of the membrane was not affected, and the gas semipermeability remained mostly preserved in all cases.

Harsh organic solvents lead to either swelling or shrinkage of the substrate fiber and sometimes irreversible changes in gas permeable characteristics of the membrane, although the integrity of the plasma polymer coating was not affected, and membranes remained permselective.

We also found that not all applications of plasma polymer-coated membranes are affected by the aging process. This is especially true of membranes used in membrane distillation and other such processes, which are governed by the pore size or surface hydrophobicity/hydrophilicity of the substrate and not so much by the membrane gas separation characteristics. Here the plasma coating either prevented fouling of the membrane surface or promoted high water vapor flux through the membrane pores. This aspect of plasma membrane application has been covered in detail in one of our recent publications [47]. Hollow fiber membranes designed for VOC removal using plasma polymerization, whether they used a closed pore structure or an open pore structure, were also not expected to be affected by the aging process. Some of these membranes have retained their overall performance over a period of one year.

## Figures and Tables

**Table 1:** Typical properties of hollow fiber membrane (HFM) substrates used.

PROPERTIES	X30-240	X30-150	PP50/200	PP50/280	PP150/330
Polymer	PP	PP	PP	PP	PP
OD (μm)	300 ± 6	200 ± 7	300 ± 20	380 ± 20	630 ± 50
ID (μm)	233–255	140–160	200	280	330 ± 50
Wall Thickness (μm)	28 ± 2	25 ± 3	50 ± 10	50 ± 10	150 ± 25
Pore Size(μm)	0.04 (0.04 × 0.10)	-	≤0.2	≤0.2	0.60
Porosity (%)	40%	40%	-	50–55%	
Resistance to Air Flow (Gurley * sec)	25–45	49–76	-	-	-
Nitrogen Permeance (cm^3^ cm^−2^ s^−1^ cm Hg^−1^)		-	1.67 ± 0.67 ×10^−2^	1.67 ± 0.67 ×10^−2^	-
Tensile Strength at Break	≥175 g/fil	≥100 g/fil	153 g/fil (150 cN) **	153 g/fil (150 cN)	-
Burst Strength	220 psi	200 psi	-	-	-
Bubble Point	-	-	45 psig in IPA ***	44 psig in IPA	15 psig in IPA
Solvent Residue	-	-	≤100 ppm	≤ 100 ppm	-
Elongation at Break	≥50%	-	400%	400%	
Explosion Pressure	-	-	44 psig	44 psig	-
Implosion Pressure	-	-	51 psig	51 psig	-
Suggested Applications	Blood Oxygenators, Gas Separation, Liquid Degassing, Biotechnology, etc.	Cardiac Therapy, Gas separation, and other Industrial applications	Biological separations, Blood Oxygenation, and other medical applications	Gas Contactors, Air filtration, Water filtration, Food and Beverage	Industrial oxygenation, Membrane Distillation, Waste water treatment, etc.

Source: 3M/Membrana Product Brochures, * Gurley Number is the time in seconds it takes for 100 cm^3^ of air to pass through one square inch of membrane when a constant pressure of 4.88 inch of water is applied, ** 1cN = 1.02 gm force, *** IPA = Isopropanol.

**Table 2:** Effect of monomer flow rate and reaction time on N_2_ permeance (PP50/280).

Sample No.	TMDSO Flow Rate (SCCM)	Reaction Time (sec)	N_2_ Permeance * (10^−3^ cm^3^ cm^−2^ s^−1^ cm Hg^−1^)	Effective Pore Diameter (μm)
Untreated PP 50/280	-	-	12.1	0.200
3069	6.1	17	11.6	0.191
3070	10.1	17	10.6	0.178
3071	14.9	17	8.3	0.164
3119	6.1	25	10.1	0.177
3120	14.9	25	4.1	0.112
3121	22.3	25	2.9	0.095

* Permeance = (N_2_ flux)/(N_2_ pressure difference).

**Table 3:** Effect of monomer flow rate and reaction time on N_2_ permeance (PP50/200).

Sample No.	TMDSO MFR (SCCM)	Reaction Time (Sec)	N_2_ Permeance (10^−3^ cm^3^ cm^−2^ s^−1^ cm Hg^−1^)	Effective Pore Diameter (μm)
Untreated PP 50/200	-	-	12.00	0.200
2394	2.0	12	10.40	0.186
2393	3.4	12	9.90	0.181
2392	6.1	12	9.30	0.176
2391	6.1	17	8.38	0.167
2390	6.1	25	4.81	0.127
2426	10.1	25	1.17	0.063
2417	12.2	25	0.70	0.048
2418	15.5	25	0.28	0.031
2420	18.3	25	0.15	0.022

**Table 4:** Effect of monomer flow rate on membrane N_2_ permeance (PP150/330).

Sample No.	TMDSO MFR (SCCM)	Reaction Time (Sec)	N_2_ Permeance (10^−3^ cm^3^ cm^−2^ s^−1^ cm Hg^−1^)	Effective Pore Diameter (μm)
Untreated PP 150/330	-	-	73.7	0.600
5803	6.1	25	61.4	0.548
5804	12.2	25	59.7	0.540
5805	22.3	25	56.2	0.524

**Table 5:** Contact angle with water of plasma polymer coatings on microglass slides.

Sample No.	PFM	θ H_2_O at t = 0	θ H_2_O at t = 3 year	θ H_2_O at t = 48 h in Water
6090	TMDSO	103°	100°	98°
6093	TMDSO/HFE	102°	101°	91°
6573	TMDSO/PFHX	105°	106°	107°
6245	HFE	32°	-	17°

**Table 6:** Tensile properties of PP50/200 HFM before and after coating with F/Si Plasma polymer as a function of W/FM.

Sample No.	TMDSO Flow Rate (SCCM)	W/FM (10^6^ J kg^−1^)	Load (Standard Deviation) (N/fiber)	Elongation (Standard Deviation) (%)
Untreated Membrane	-		1.58 (1.77)	578.1 (30.05)
#2390	6.1	132.2	1.53 (3.79)	637.5 (16.58)
#2395	8.1	99.8	1.56 (3.26)	659.5 (15.21)
#2396	10.2	78.2	1.59 (2.89)	617.5 (18.76)
#2401	14.3	56.6	1.54 (2.89)	571.5 (26.26)

**Table 7:** Tensile Properties of X30-240 HFM before and after coating with a siloxane plasma polymer.

Sample No.	TMDSO Flow Rate (SCCM)	W/FM (10^6^ J kg^−1^)	Load (Standard Deviation) (N/fiber)	Elongation (Standard Deviation) (%)
Untreated X30-240	-	-	2.35 (0.04)	117.7 (7.0)
2285 *	14.85	47.2	3.63 (0.01)	76.0 (6.0)
2286 **	14.85	47.2	3.62 (0.01)	73.9 (7.3)
2287 **	14.85	53.9	3.53 (0.02)	70.0 (5.3)

* Residence time: 10 s ** Residence time: 12.5 s.

**Table 8:** Effect of monomer type on water flux in DCMD at feed brine concentration of 8% *w*/*v*.

Sample No.	PFMs (Monomers)	Brine Temp. (°C)	Cold Water Temp. (°C)	Water Flux (kg m^−2^ h^−1^)	Product Water Conductivity (μS cm^−1^)
Uncoated		70	23	11.9	3–4
6108	TMDSO/HFE	70	23	13.4	5–10
6179	TMCTS/HFE	70	23	15.2	4–9
6199	TMDSO/PFFM	70	23	17.5	4–8
6199 *	TMDSO/PFFM	70	23	17.0	2–4
6199 **	TMDSO/PFFM	70	23	18.2	2–3

* 2 months old ** 3 months old and used.

**Table 9:** Effect of brine water temperature on water flux for a brine concentration of 8% *w*/*v*.

Sample No.	PFMs	Brine Temp. (°C)	Cold Water Temp. (°C)	Water Flux (kg m^−2^ h^−1^)	Product Water Conductivity (μS cm^−1^)
6199	TMDSO/PFFM	50	23	7.4	1.9–2.2
6199	TMDSO/PFFM	70	23	18.3	1.8–2.1
6179	TMCTS/PFFM	81	23	55.6	2.1–2.4

**Table 10:** Effect of aging in air on CO_2_ gas permeance for membranes made from TMDSO, TMCTS, TMSAA, and HMTSO polymers (substrate X30-240).

Sample No.	PFM	Decrease in CO_2_ Permeance at Different Time Intervals				
		7 days	14 days	28 days	60 days	120 days
5978	TMDSO	9%	11%	15%	18%	22%
6244	TMCTS	6%	10%	17%	28%	
5979/5978 *	TMSAA	3%	5%	7%	10%	
6081	HMTSO	2%	3%	5%	-	7%

* TMSAA plasma polymer overcoat.

**Table 11:** Changes in the gas permeability characteristics of TMDSO polymer-coated X30-150 HFM in the bulk of the spool.

Sample No.	Age of Sample (Day)	Gas Permeance (10^−4^ cm^3^ cm^−2^ s^−1^ cm Hg^−1^)			Selectivity
		N_2_	O_2_	CO_2_	CO_2_/O_2_
2991	1	1.86	3.77	14.8	3.94
2991 *	3405	1.52	3.13	13.1	4.19
2991 **	3405	1.65	3.45	14.2	4.10

* After removing 500 m fiber from top of coated spool ** After complete rewinding of the spool.

**Table 12:** Changes in the gas permeability characteristics of TMDSO polymer-coated X30-240 HFM on spool.

Sample No.	Age of Sample (Day)	Gas Permeance (10^−4^ cm^3^ cm^−2^ s^−1^ cm Hg^−1^)			Selectivity
		N_2_	O_2_	CO_2_	CO_2_/O_2_
2985	1	1.74	3.36	13.10	3.90
2985 *	515	1.86	3.06	10.20	3.32
2985 **	4650	1.67	2.75	11.0	3.96
2985 ***	4830	1.67	2.74	10.3	3.75

* HFM aged on shepherd hooks ** After removing 500 m fiber from top of coated spool *** Shepherd hooks prepared after 4650 days and re-aged for 6 more months.

**Table 13:** Atomic concentrations in atomic percent of elements in the surface of TMDSO and F/Si plasma polymer coated X30-240 HFM.

Sample No.	PFM	Age of Sample	%C	%Si	%O	%F	C/Si	O/Si Ratio
6348	TMDSO	33 Days	38.39	27.68	33.92	-	1.39	1.225
6348	TMDSO	52 Days	38.04	27.07	34.89	-	1.40	1.289
3175-6	TMDSO/HFE	320 Days	33.30	19.50	31.50	15.7	1.71	1.615
7905 *	-	-	100.0	-	-	-	-	-

* Untreated PP150/330.

**Table 14:** Effect of heat aging at 90–100 °C for 90 min on gas permeance of membranes made from TMDSO and HMTSO plasma polymers.

Sample No.	PFM	RT (sec)	CO_2_ Gas Permeance (10^−4^ cm^3^ cm^−2^ s^−1^ cm Hg^−1^)		% Change	Selectivity CO_2_/O_2_	
			Before	After		Before	After
6620	TMDSO	14	6.48	5.53	(−14.7%)	4.40	4.60
6621	TMDSO	20	4.31	3.39	(−21.4%)	4.85	5.63
7011	HMTSO	12	24.1	17.1	(−29.0%)	4.25	4.45
7011	HMTSO	12	24.1	20.1 *	(−16.6%)	4.25	4.45

* After taking the length shrinkage into account.

**Table 15:** Change of gas permeability characteristics of TMCTS plasma polymer coated X30-240 fiber on exposure to 0.9% buffered saline.

Exposure Time	Sample No.	Gas Permeance and Selectivity before Exposure (10^−4^ cm^3^ cm^−2^ s^−1^ cm Hg^−1^)				Gas Permeance and Selectivity after Exposure (10^−4^ cm^3^ cm^−2^ s^−1^ cm Hg^−1^)			
		N_2_	O_2_	CO_2_	CO_2_/O_2_	N_2_	O_2_	CO_2_	CO_2_/O_2_
7 days	6258-7A	2.30	4.00	15.70	3.93	1.70	3.26	13.99	4.29
	6258-7B	2.47	4.09	15.40	3.77	1.81	3.33	13.96	4.19
	6258-7C	2.36	4.08	16.16	3.96	1.90	3.48	14.63	4.20
14 days	6258-14A	2.47	4.18	16.27	3.89	1.68	3.27	14.24	4.35
	6258-14B	2.12	3.83	15.90	4.15	1.54	3.12	14.00	4.49
	6258-14C	2.38	4.10	16.35	3.98	1.75	3.33	14.37	4.31
21 days	6258-21A	2.32	4.00	15.86	3.96	1.56	3.10	13.57	4.38
	6258-21B	2.34	4.03	15.87	3.94	1.63	3.17	13.76	4.34
	6258-21C	2.47	4.21	16.25	3.86	1.63	3.19	14.07	4.41
28 days	6258-28A	2.31	4.11	16.61	4.05	1.37	2.81	12.75	4.53
	6258-28B	2.43	4.20	16.53	3.94	1.42	2.85	12.62	4.42
	6258-28C	2.18	3.94	14.79	3.76	1.38	2.82	12.28	4.36

**Table 16:** Change of gas permeability characteristics of TMDSO plasma polymer coated X30-150 fiber on exposure to 0.9% buffered saline.

Exposure Time	Sample No.	Gas Permeance and Selectivity Before Exposure (10^−4^ cm^3^ cm^−2^ s^−1^ cm Hg^−1^)				Gas Permeance and Selectivity After Exposure (10^−4^ cm^3^ cm^−2^ s^−1^ cm Hg^−1^)			
		N_2_	O_2_	CO_2_	CO_2_/O_2_	N_2_	O_2_	CO_2_	CO_2_/O_2_
7 days	6281-7A	0.66	1.78	8.44	4.74	0.68	1.64	7.63	4.65
	6281-7B	0.68	1.83	8.67	4.73	0.64	1.64	7.66	4.68
	6281-7C	0.72	1.90	9.07	4.78	0.84	1.90	8.16	4.31
14 days	6281-14A	0.67	1.85	9.00	4.86	0.64	1.64	7.62	4.65
	6281-14B	0.67	1.79	8.37	4.67	0.57	1.51	7.21	4.78
	6281-14C	0.66	1.78	8.30	4.67	1.37	2.29	7.66	3.35
21 days	6281-21A	0.66	1.78	8.46	4.75	0.70	1.65	7.30	4.41
	6281-21B	0.64	1.71	8.31	4.85	0.64	1.56	7.17	4.60
	6281-21C	0.72	1.89	8.94	4.73	0.89	1.87	7.96	4.25
28 days	6281-28A	0.70	1.86	8.89	4.79	0.74	1.70	7.52	4.42
	6281-28B	0.66	1.79	8.67	4.83	0.61	1.61	7.53	4.68
	6281-28C	0.68	1.81	8.74	4.82	0.57	1.44	6.78	4.69

**Table 17:** Changes in gas permeability characteristics on exposure to chemicals.

Sample No.	Chemicals	Gas Permeance and Selectivity before Exposure (10^−4^ cm^3^ cm^−2^ s^−1^ cm Hg^−1^)				Gas Permeance and Selectivity after Exposure (10^−4^ cm^3^ cm^−2^ s^−1^ cm Hg^−1^)			
		N_2_	O_2_	CO_2_	CO_2_/O_2_	N_2_	O_2_	CO_2_	CO_2_/O_2_
5229-4	NH_4_OH 0.45 M	1.07	2.35	9.62	4.09	0.98	2.19	8.91 (−7.90%)	4.06 (−0.7%)
5229-5	NH_4_OH 0.90 M	1.08	2.37	9.68	4.08	0.96	2.18	8.96 (−7.40%)	4.10 (+0.5%)
5229-9	DEA 20% H_2_O	1.12	2.36	9.39	3.97	1.06	2.15	8.27 (−12.0%)	3.85 (−3.0%)
5229-6	TOA 50% EtOH	1.05	2.26	9.16	4.06	0.63	1.94	7.43 (−18.9%)	3.83 (−5.6%)
						0.66 *	1.92 *	7.99 * (−16.8%)	4.17 * (+2.7%)
5229-7	DC 200 50% EtOH	1.02	2.28	9.23	4.05	0.94	2.04	7.85 (−15.0%)	3.85 (−5.0%)
5229-8	Hexane	1.02	2.28	9.36	4.11	0.85	2.11	7.91 (−15.5%)	3.75 (−8.7%)
AJ1033	2-PrOH	4.37	5.48	13.7	2.50	3.38	4.59	13.3 (−3.0%)	2.89 (+15.6%)
AJ1031	DCM	4.58	5.71	14.2	2.48	2.65	3.67	11.2 (−22.2%)	3.04 (+22.6%)

* Results after further drying for 24 h.

**Table 18:** Effect of composite parameter W/FM on change in gas permeability characteristics after solvent exposure.

Sample No.	W/FM (10^6^ J kg^−1^)	Chemicals	Gas Permeance and Selectivity before Exposure (10^−4^ cm^3^ cm^−2^ s^−1^ cm Hg^−1^)				Gas Permeance and Selectivity after Exposure (10^−4^ cm^3^ cm^−2^ s^−1^ cm Hg^−1^)			
			N_2_	O_2_	CO_2_	CO_2_/O_2_	N_2_	O_2_	CO_2_	CO_2_/O_2_
5292-2	94.4	Hexane	0.47	1.22	5.76	4.73	0.35	0.93	4.42 (−23%)	4.74 (+0.2%)
5300-1	47.2	Hexane	0.76	1.61	6.70	4.16	0.35	0.93	4.40 (−34%)	4.74 (+5.8%)
5292-1	94.4	Toluene	0.47	1.22	5.79	4.75	0.28	0.81	3.84 (−33%)	4.72 (+0.6%)
5300-3	47.2	Toluene	0.75	1.58	7.01	4.44	0.46	1.00	4.36 (−38%)	4.38 (−1.4%)
